# Love Wave Immunosensor for the Detection of Carbaryl Pesticide

**DOI:** 10.3390/s140916434

**Published:** 2014-09-03

**Authors:** María-Isabel Rocha-Gaso, José-Vicente García, Pablo García, Carmen March-Iborra, Yolanda Jiménez, Laurent-Alain Francis, Ángel Montoya, Antonio Arnau

**Affiliations:** 1 Grupo de Fenómenos Ondulatorios (GFO), Universitat Politècnica de València, 46022 Valencia, Spain; E-Mails: marocga@doctor.upv.es (M.-I.R.-G.); jogarnar@upvnet.upv.es (J.-V.G.); pablo@awsensors.com (P.G.); yojiji@eln.upv.es (Y.J.); 2 Sensors, Microsystems and Actuators Laboratory of Louvain (SMALL), ICTEAM Institute, Université Catholique de Louvain, 1348 Louvain-la-Neuve, Belgium.; E-Mail: laurent.francis@uclouvain.be; 3 Instituto Interuniversitario de Investigación en Bioingeniería y Tecnología Orientada al Ser Humano (I3BH), Universitat Politècnica de València, 46022 Valencia, Spain; E-Mails: cmarch@eln.upv.es (C.M.-I.); amontoya@ginmuno.i3bh.es (A.M.)

**Keywords:** biosensor, carbaryl pesticide detection, immunosensor, Love Wave sensor, Surface Acoustic Wave (SAW)

## Abstract

A Love Wave (LW) immunosensor was developed for the detection of carbaryl pesticide. The experimental setup consisted on: a compact electronic characterization circuit based on phase and amplitude detection at constant frequency; an automated flow injection system; a thermal control unit; a custom-made flow-through cell; and Quartz/SiO_2_ LW sensors with a 40 μm wavelength and 120 MHz center frequency. The carbaryl detection was based on a competitive immunoassay format using LIB-CNH45 monoclonal antibody (MAb). Bovine Serum Albumin-CNH (BSA-CNH) carbaryl hapten-conjugate was covalently immobilized, via mercaptohexadecanoic acid self-assembled monolayer (SAM), onto the gold sensing area of the LW sensors. This immobilization allowed the reusability of the sensor for at least 70 assays without significant signal losses. The LW immunosensor showed a limit of detection (LOD) of 0.09 μg/L, a sensitivity of 0.31 μg/L and a linear working range of 0.14–1.63 μg/L. In comparison to other carbaryl immunosensors, the LW immunosensor achieved a high sensitivity and a low LOD. These features turn the LW immunosensor into a promising tool for applications that demand a high resolution, such as for the detection of pesticides in drinking water at European regulatory levels.

## 1. Introduction

Pesticides are biocides by definition, and, thus, they are potentially harmful for humans and the environment [1]. In recent years, increasing awareness about the presence of pesticide residues in the environment and food has led to an intensified search for more simple [2], rapid, selective, sensitive, power-efficient, portable, and cost-effective detection methods.

Piezoelectric immunosensors represent an attractive alternative, in terms of cost and time saving, to advanced optical devices [3] and chromatographic analysis [2]. Immunosensors do not require well-equipped centralized laboratories, they allow the use of unpurified samples, and they can be reused for multiple assay cycles. Moreover, they can be characterized with low-cost and compact electronic setups, which provide a high integration capability and in real-time analysis. The direct detection of piezoelectric immunosensors, where no labeling is required, is another significant advantage. All these features make piezoelectric immunosensors fast and economic detection methods that could save valuable time and resources for in liquid sample analysis.

The operation principle of piezoelectric immunosensors consists on immobilizing an immunoreagent on the surface of an acoustic transducer. When a direct physical change is produced in the surface due to immunochemical interactions, the acoustic wave generated by the transducer is perturbed, which causes a change in the transducer's electric signal.

Traditionally, the most commonly employed piezoelectric immunosensors are based on Quartz Crystal Microbalance (QCM) devices. This is primarily due to the fact that the QCM has been studied in detail for over more than 50 years and has become a mature, commercially available, robust and affordable technology [4]. However, Love Mode (LW) acoustic wave sensors have attracted a great deal of attention in the scientific community during the last decades due to their higher sensitivity in liquid media compared to traditional QCM-based sensors [5].

**Figure 1.**
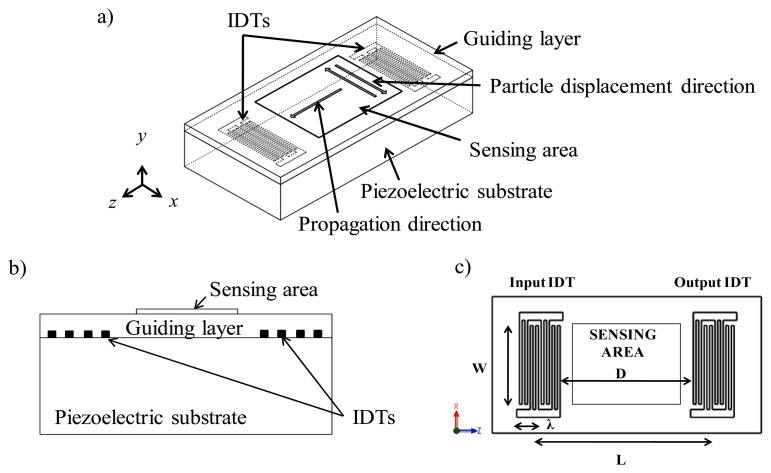
Basic structure of a LW delay line device: (**a**) Isometric view; (**b**) Cross-sectional view; and (**c**) Scheme of a LW delay line (upper view), where *W* is the acoustic aperture, *D* is the distance between input and output IDTs, *L* is the center-to-center distance between IDTs and *λ* is the acoustic wave wavelength set by the IDTs pattern periodicity.

The LW sensor is a layered structure formed by a piezoelectric substrate and a guiding layer with Interdigital Transducers (IDTs) sandwiched between the piezoelectric substrate and the guiding layer (Figure 1a,b). These devices belong to the family of Surface Acoustic Wave (SAW) devices in which the acoustic wave propagates along a single surface of the substrate. The acoustic energy of LW sensors is confined in the guiding layer [6] keeping the wave energy trapped tightly nearer the surface (Figure 1a), which makes LW devices very sensitive towards changes occurring on their sensing area [7].

Generally, LW sensors are characterized using Network Analyzers or Vector Voltmeters. However, such instruments have a high cost and considerable volume. A novel, compact and low cost electronic characterization technique based on phase and amplitude detection at fixed frequency was recently proposed and validated for QCM and High Fundamental Frequency (HFF)-QCM sensors [8,9]. In this work, the adaptation of this same characterization system for LW sensors is presented. Furthermore, custom-made LW sensors and a microsystem for in liquid measurements with the sensors were fabricated. Finally, the detection of carbaryl pesticide with an immunosensor was made possible as a direct result of these novel developments.

Carbaryl, an acetyl-cholinesterase inhibitor, is a broad-spectrum *N*-methyl carbamate insecticide [10], with low molecular weight, that has been widely used around the world [1]. Even though some adverse effects of carbaryl have been reported, it is considered a safe insecticide because of its low toxicity in mammals [10]. In this work, carbaryl was utilized as a model analyte, due to the fact that this compound has been broadly employed as the target analyte in diverse detection technologies. Therefore, a reliable performance comparison, which is presented in the discussion of this paper, can be established between the developed LW immunosensor and these other technologies.

### Operation Principle

The input IDT of a LW delay line (Figure 1b) is excited electrically applying a *radio frequency* signal. The electrical excitation generates a mechanical acoustic wave into the piezoelectric material, which is guided through the guiding layer until reaching the output IDT, where it gets transformed back to a measurable electrical signal. From an electric point of view, a LW delay line can be defined by its transfer function *H*(*f*) = *V_out_/V_in_*, which represents the relationship between input and output electrical signals. *H*(*f*) is a complex number which can be defined as *H*(*f*) = *Ae^jφ^*, being *A* = |*V_out_/V_in_*| the *amplitude* and *φ* is the *phase* of *V_out_/V_in_*. In addition, the insertion loss (IL) is generally defined from the amplitude as 20log_10_(*A*). Immunochemical interactions where antibodies bind to target analytes can be mainly detected as mass variations at the sensor surface. These interactions produce changes in the amplitude and velocity of the propagating surface acoustic wave, which in turn are translated to changes in the electrical amplitude and phase of the sensor response.

## 2. Materials and Methods

### 2.1. Fabrication of LW Sensors and Flow Cell

**Figure 2.**
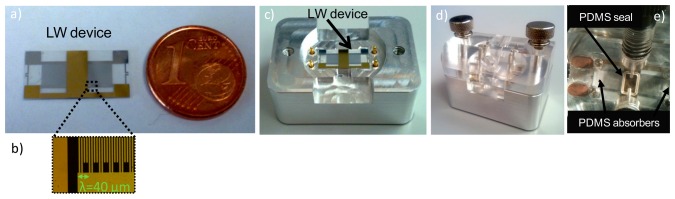
(**a**) Size comparison of the final fabricated LW device with a one centime euro coin; (**b**) Microscope picture of the IDTs fingers; (**c**) Lower part of the flow cell with sensor mounted; (**d**) Custom-made flow cell; (**e**) The sensing area sealing and PDMS absorbers seen from a microscope through the upper part of the flow-through cell.

LW delay lines were fabricated on 17 × 8.4 mm and 0.35 mm thick single-side polished Z-propagating AT-cut quartz substrates. The input and output IDT consisted of 100 double-finger pairs with 5 μm width and 5 μm separation (leading to a wavelength *λ* = 40 μm). The IDTs aperture, *W*, was 3.5 mm and the IDTs center-to-center distance, *L*, was 7.48 mm. A 3 μm guiding layer of silicon dioxide (SiO_2_) was deposited on top of the devices via Plasma Enhanced Chemical Vapor Deposition (PECVD). Finally, the sensing area was deposited in the space between the IDTs by evaporation and lift-off of 10 nm of chrome and 50 nm of gold. Such structure led to devices with center frequency around 120 MHz. Figure 2a presents the final fabricated device.

We fabricated a custom-made flow cell, which allowed the connection of the device with the characterization equipment (see Figure 2c,d). The liquid sealing was achieved with a rectangular-shape Polydimethylsiloxane (PDMS) seal, which delimited a sensing area of 2.4 × 3.8 mm dimensions [11]. PDMS acoustic absorbers were also employed at the end of each IDT (see Figure 2e). Such structures were 3.8 × 1 × 1 mm PDMS rectangular prisms, which helped to enhance the sensor frequency response “cleaning it”, by avoiding unwanted reflections at the sensors' edges. The lower part of the cell was made of aluminum, which was electrically connected to the electronic ground, in order to generate a metal shielding that protected the sensor from electromagnetic external effects. The cell dimensions were 3.3 cm wide by 4.7 cm long by 3.1 cm high. Just in the bottom of the lower part of the cell, an interface printed circuit board (PCB) was located that was used to place some components of the electronic characterization system and to connect the sensor to the circuit. The sensor connection with the cell was carried out with spring contacts, thus, no wire-bonding was required, ensuring a simple and rapid removal and replacement of the sensors. In addition, the sensing area of the LW sensors was grounded so the effects of electrical fields could be neglected, letting the device to be sensitive mainly to mechanical perturbations [12].

### 2.2. Network Analyzer

Network analyzer measurements were carried out using a two-port Rohde and Schwarz ZV24 Network Analyzer, which had a frequency range from 10 MHz to 24 GHz and a maximum number of test points per trace of 60001.

### 2.3. Electronic Instrumentation Measurement Technique and Experiment Setup

The characterization technique employed in this work, was adapted from a phase and amplitude characterization technique at a fixed frequency previously described and validated for QCM sensors [9]. This technique met the following requirements: (1) its stability and the minimum signal that it can measure maximizes the signal to noise ratio, thus, mass resolution is enhanced; (2) it provides the electric parameters of interest for biosensing applications: phase and IL. Phase is directly related to mass changes at the sensor's surface and the ratio IL/ΔPhase provides valuable information regarding the viscoelastic properties at the liquid interface; (3) it is fast enough to allow the characterization of multiple sensors in a reasonable time; and (4) its high integration capability and low cost make it suitable for its use out of centralized laboratories.

**Figure 3.**
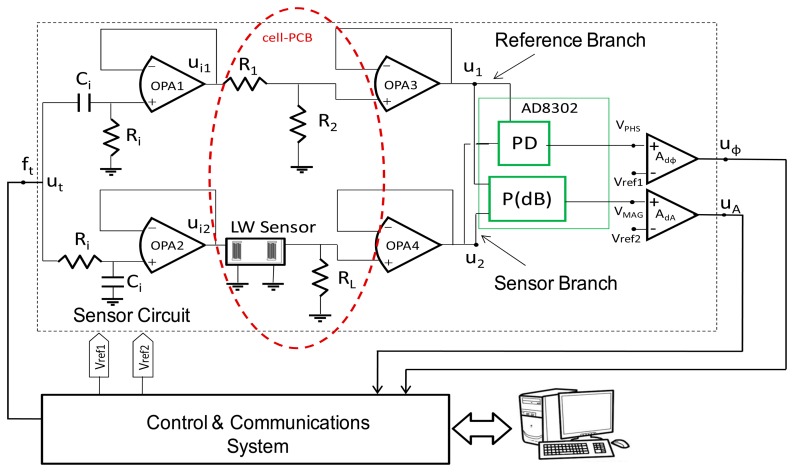
Schematics of the phase and amplitude electronic characterization system employed for the LW sensor.

Figure 3 shows a block diagram of the electronic set up. Two parts can be distinguished in the circuit: the Sensor Circuit (inside the dashed rectangle line)—which includes the sensor—and the Control and Communication System.

Two parallel branches form the Sensor Circuit, the Sensor Branch (containing the sensor) and the Reference Branch, creating a differential circuit. The circuit elements inside the elliptical dotted line, differ from the ones described in reference [9] and are placed in the LW flow-through cell PCB. The rest of components remain unchanged, hence, the same characterization system could be used to characterize both, QCM and LW devices. Both branches are excited with the same test signal *u_t_* generated by a direct digital synthesis. The signal *u_t_* has a constant frequency, and constant amplitude, therefore, the reference branch produces a constant signal *u*_1_, which serves as a reference signal. On the other hand, when a perturbation takes place on the surface of the sensor, a change in the phase velocity and energy of the acoustic wave is produced, generating a change in the electrical signal at the sensor's output port. The changes in the amplitude and phase of the sensor branch signal *u*_2_, relative to the unchanged reference signal *u*_1_, due to perturbations, are provided by the AD8302 Integrated Circuit (IC). In order to have the maximum linear range provided by this IC the signals *u*_1_ and *u*_2_:1) must be of similar amplitudes and 2) must be phase-shifted 90°. To meet requirement 1, the voltage divider formed by *R*_1_ and *R*_2_ in the reference branch was designed to produce a similar IL to the one provided by the sensor branch when the sensor is in contact with the sample buffer, PBS (10 mM phosphate buffer). With *R*_1_ = 560 Ω and *R*_2_ = 49.9 Ω the IL were about −22 dB. To meet requirement 2, the phase shifting networks formed by *R_i_* and *C_i_* were employed [9]. The values of *R_i_* and *C_i_* were coherently designed for a cut-off frequency (−3dB) of 120 MHz (ω*_c_* = 1\*R_i_C_i_*), being 49.9 Ω (1%) and high−Q 25 pF for *R_i_* and *C_i_*, respectively.

*R_L_* is required to polarize amplifier OPA4 and its value was chosen to meet the maximum transfer power, considering that the working conditions of the sensor are the ones corresponding to the sensor mounted in the cell and loaded with the sample buffer, PBS [11].

The Control and Communication System controls the test signal generation, and the signals, *u_A_* and *u_ϕ_*, conversion and acquisition. The entire system provided 620.3 mV/° for the phase signal and 1869 mV/dB for the amplitude signal.

**Figure 4.**
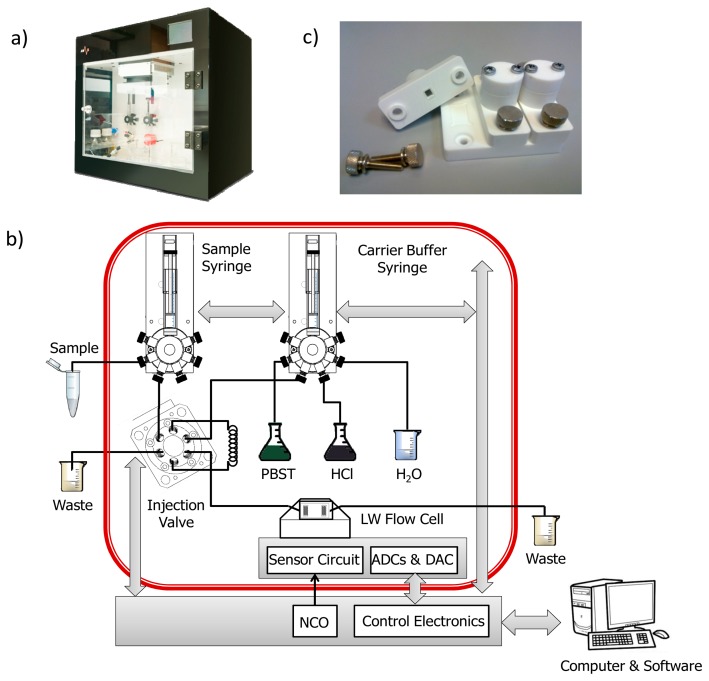
(**a**) A10 test platform (developed in collaboration with AWSensors Corp.). The platform incorporates: the fabricated LW flow cell; a flow circuit with automated syringe pumps, distribution and injection valves; a thermal control unit; and the electronic characterization system based on a phase and amplitude measurement at constant frequency (taken with permission from www.awsensors.com); (**b**) Final experimental setup built with the A10 platform; (**c**) Fabricated immobilization cells for LW devices.

Figure 4a shows the photograph of the employed A10 test platform, which was developed in collaboration with the company AWSensors Corp., Spain. The platform integrates: (1) the electronic characterization system previously described; (2) the fabricated flow cell were the immobilized sensors were placed; (3) the flow-through system, that consisted of distribution\injection valves and syringes arranged to create an automated flow injection analysis system described elsewhere [13], which allowed buffers and samples to flow onto the sensing area of the sensors; and (4) the thermal control unit that allowed to perform experiments at a stable temperature of 25 °C ± 0.1 °C.

The A10 test platform was connected with an Ethernet cable to an external computer where the acquisition software was run. Figure 4b depicts the final experimental setup. In this figure, the elements delimited by the red line were located inside the thermostatic chamber of the platform.

The AWS-BIO v1.8 (AWSensors Corp., Spain) software was employed to: control the flow-through system; set the operation frequency; and to display and record all the measurements. Thus, the phase, amplitude and temperature were continuously monitored during the immunoassays experiments. In addition, the software allowed two different operation modes of the electronic characterization system: a *High Resolution Mode* and a *Sweep Mode*. In the High Resolution Mode, the signal *u_t_* has a fix frequency, *f_t_*. In this operation mode, the system acquires the voltages *u_φ_* and *u_A_* at *f_t_* in real-time. In the Sweep Mode, the frequency of the signal *u_t_* is varied in a frequency range selected by the user. In this way, the system acquires the voltages *u_φ_* and *u_A_* for such frequencies.

### 2.4. Immunosensors

#### 2.4.1. Chemicals and Immunoreagents

All the employed chemicals and immunoreagents were of analytical grade and the same as described in reference [2], except for the mercaptohexadecanoic acid (MHA) which was used instead of thioctic acid for the formation of the self-assembled monolayer. MHA was supplied by Sigma-Aldrich Chemie (Madrid, Spain). The immunoreagents were produced by the Immunotechnology Group of I3BH, Polytechnic University of Valencia, Spain. The production of the employed immunoreagents was described in other works [10,14]. Previous studies determined that the conjugate-MAb combination for carbaryl pesticide detection that provides the best assay performance and regeneration capability for piezoelectric sensors was the LIB-CNH45 MAb with BSA-CNH hapten-conjugate [2]. Therefore, we employed such immunoreagents in this work.

#### 2.4.2. Immunoassay Format

Given that carbaryl pesticide is a small analyte, the immunosensor was developed in a competitive inhibition format. Since the antibody immobilization often leads to impaired regeneration capability and poor immunoassay reproducibility of immunosensors, a conjugate-coated assay format was chosen. This way, the performance of the immunoassay in terms of stability and reliability was improved [2].

#### 2.4.3. Covalent Immobilization

##### LW Sensors Clean Up

LW sensors were first cleaned by immersion into acetone and ethanol, followed by subsequent rinses with double distilled water. Afterwards, the sensors were blown dry with a stream of nitrogen gas. Once dried, the sensors were exposed to UV rays and ozone during 30 min using the ProCleaner™ (BioForce Nanosciences Inc., IA, USA). This step was carried out in order to remove contamination at molecular level and obtain the cleanest sensing area possible. After this, the sensors were cleaned with double distilled water, ethanol, and, subsequently, were dried with nitrogen gas.

##### Covalent Immobilization via Mercaptohexadecanoic Acid Self-Assembled Monolayers (SAM)

Specific immobilization cells for the LW devices were fabricated to carry out the immobilization processes (Figure 4c). Covalent immobilization via mercaptohexadecanoic acid Self-Assembled Monolayers (SAM) was performed. The same immobilization procedure, described in reference [2], was followed, except for the volume and concentration of the thiolated compound solution; 500 μL of 50 μM mercaptohexadecanoic acid ethanolic solution was employed. This immobilization technique ensures highly ordered protein immobilization, which provides numerous advantages, as the improvement of detection limits, reproducibility, reusability and prevention of non-specific binding of biomolecules [2]. In the hapten-conjugate immobilization step, 120 μL of BSA-CNH hapten-conjugate in 0.1 M sodium phosphate buffer, pH 7.5, was placed on the gold sensing area for 4 h 30 min. The rest of the procedure remained the same as the one reported in reference [2], but with solution volumes of 120 μL instead of 60 μL, due to the larger sensing area of LW sensors.

##### Immunoassay Protocol and Standard Calibration Curves

The immunoassay protocol was the following: (1) 5 min flow of PBST at a flow rate of 20 μL/min to stabilize the baseline signal; (2) Sample injection (350 μL) during 15 min at the same flow rate; and (3) 4 min regeneration with HCl and 4 min with PBST at a flow rate of 250 μL/min. Thus, a complete assay cycle took in total 28 min. After 15 min of sample injection, the signal was stabilized and the increment in phase with respect to the one at the time of sample injection (baseline signal) was measured: Δ*u_φ_* = *u_φ_* − *u_φ_*_0_.

Standard solutions of carbaryl, in the range of 10^−4^ to 10^3^ μg/L, were prepared, from 1 mM stock solution, by serial dilutions in 10 mM phosphate buffer (PBS) with pH 7.45 and stored at −20 °C in dark vials. The standards were mixed with a fixed concentration of LIB-CNH45 MAb. Analyte-antibody solutions were incubated for 1 h at room temperature and then injected and brought onto the sensor surface. The phase and amplitude of the LW sensor was monitored in real-time as the binding between free antibody and the immobilized hapten-conjugate took place. Regeneration of the functionalized surface was accomplished with 0.1 M hydrochloric acid (HCl) to break the antibody-hapten-conjugate association.

Standard curves were obtained by plotting the voltage phase increment *vs*. the logarithm of analyte concentration. The curves were normalized by expressing the Δ*u_φ_* obtained for each standard concentration as a percentage of the maximum response (maximum signal at 0 analyte concentration, Δ*u_φmax_* = 100%); that is to say: 100·Δ*u_φ_*/Δ*u_φmax_*. All standards and samples were run at least three times to obtain their respective means and standard deviation errors.

The experimental points were fitted to the four-parameter logistic equation:

(1) $$y=D+\frac{\left(A-D\right)}{1+{\left(\overset{x}{\;}/\underset{C}{\;}\right)}^{B}}$$

where *A* is the asymptotic maximum (maximum signal in the absence of analyte, Δ*u_φmax_*), *B* is the curve slope at the inflection point (related to the analyte concentration giving 50% inhibition: *C*, *I*_50_) and *D* is the asymptotic minimum (background signal).

### 2.5. Selection of the Optimal Operating Frequency

In order to choose the optimal operating frequency, *f_op_*, of the sensor in the system, frequency sweeps were carried out with the Platform Sweep Mode in a suitable frequency range while the immobilized sensors were loaded with the working buffer, PBST (10 mM phosphate buffer containing 0.005% Tween 20), at a flow speed of 20 μL/min. The chosen sensor *f_op_* was the one at which the amplitude of the sensor frequency response was maximum and at which the phase was 0°. This was done to work at an effective range of linear phase and at the better sensor performance. Once the optimal frequency was selected, the real-time acquisition was started at that fixed frequency with high frequency stability.

## 3. Results and Discussion

### 3.1. Response of the LW Sensor Mounted in the Flow-Through Cell

**Figure 5.**
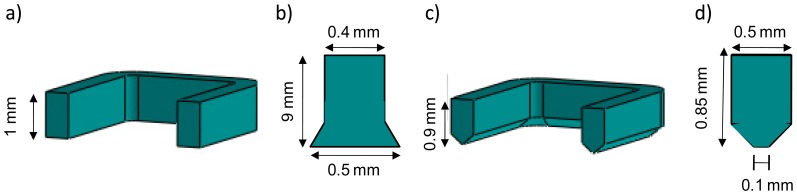
Schematics of the tested PDMS central seals. (**a**) Cross-sectional view of the first tested rectangular seal with a flat end; (**b**) 2D view of one of the flat end seal's walls pressed onto the sensor surface; (**c**) Cross sectional view of the rectangular seal with a peak end; (**d**) 2D view of one of the peak-end seal's walls pressed onto the sensor surface.

**Figure 6.**
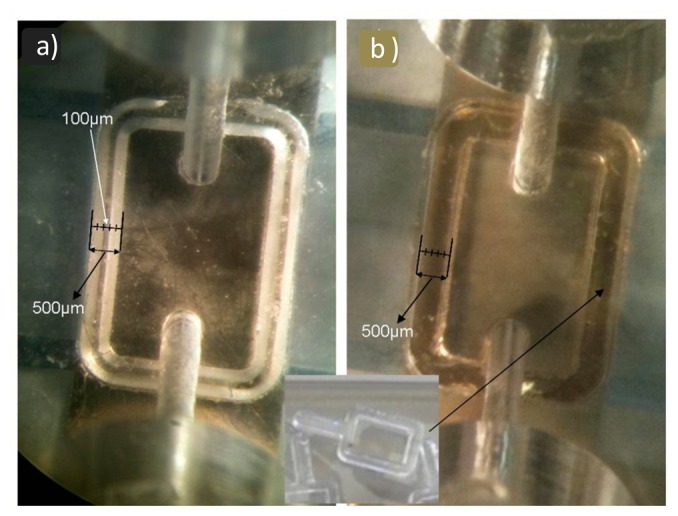
Real view of PDMS rectangular seals seen inside the cell through the transparent PMMA using an optical microscope. (**a**) Rectangular seal with peak-end; (**b**) Rectangular seal with flat-end.

**Figure 7.**
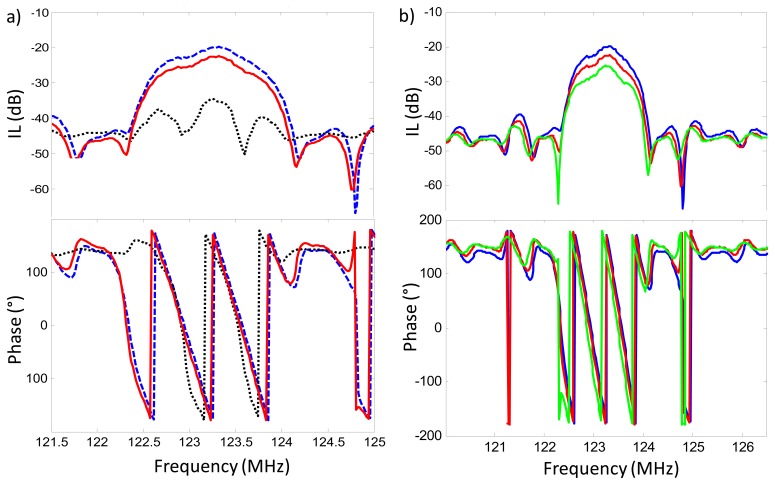
Comparison between different frequency responses of the same fabricated LW sensor mounted in the flow-through cell. The measurements were obtained using a Network Analyzer. The insertion loss and the phase have been depicted in the upper and lower part of the figures, respectively. (**a**) Sensor in contact with air. The dotted, solid, and dashed responses were obtained with the flat-end seal, the peak-end seal and no seal on the sensing area, respectively; (**b**) Blue solid line represents the sensor response with no central seal; red solid line with the central peak-end seal; and green solid line with the peak-end seal and a double distilled water load.

The first rectangular seal we used was of dimensions 0.4 mm width and 1 mm high and had a flat end (see Figure 5a). When placing the upper part of the cell and applying some pressure onto the seal, the area in contact with the LW sensor's surface became greater (see Figure 5b). We estimated that the thickness of the wall in contact with the sensor was of approximately 0.5 mm with this seal (see Figure 6b). When such thick walls were introduced on the way of the propagating acoustic wave, the sensor's frequency response was greatly distorted and attenuated (see dotted line in Figure 7a). This result led us think for a different approach to minimize the contact area of the seal on the sensor's surface. A better sensor response was achieved using a rectangular seal with a peak end (see Figure 5c), which minimized the contact area on the sensor surface (see Figure 6a). The brightest zones in Figure 6 are the areas of the seals in contact with the sensor. The reader can appreciate in the figures how the peak-end seal reduces the area in touch with the sensor diminishing the distortion of the sensor response. The sensor response obtained with the rectangular peak-end seal is presented in the solid line of Figure 7a. As it can be appreciated in this last figure, the sensor response with the peak-end seal approaches the one obtained with no seal on the sensing area (dashed line in Figure 7a) and a lower attenuation and distortion is produced on the sensor response compared to the one obtained with the flat-end seal. Finally, the green solid line in Figure 7b shows the sensor response with the peak-end seal and a double distilled water load.

**Figure 8.**
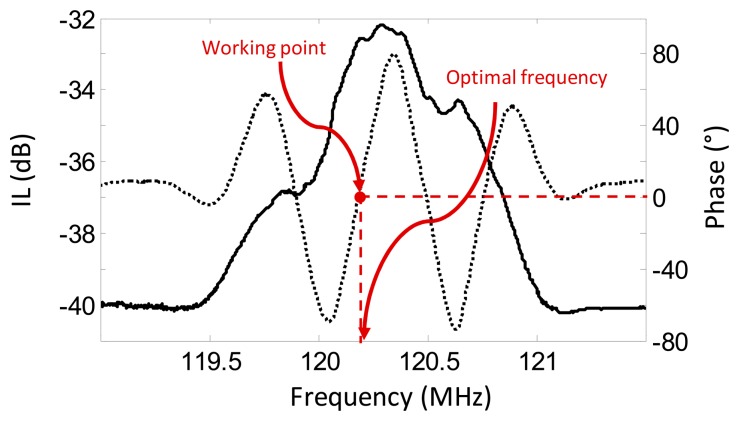
Frequency response of the LW sensor mounted in the fabricated flow-through cell and loaded with water. The measurement was obtained with the electronic characterization system. The solid line represents the IL loss and the dotted line represents the phase. The red spot indicates the working point that it is used for determining the optimal operation frequency of the sensor.

Figure 8 shows the frequency response of the LW sensor mounted in the flow cell loaded with double distilled water, obtained with the Sweep Mode of the electronic characterization system previously described. A minimum IL of approximately −28 dB was observed at the device center frequency of approximately 120 MHz. The response of the sensor was not greatly distorted and the phase was linear around the working point. Thus, the fabricated flow-through cell allowed an effective operation of the LW sensors in contact with liquid media.

### 3.2. LW Carbaryl Immunoassay Optimization

For competitive immunoassays the selection of suitable MAb and hapten-conjugate concentrations are crucial to assure the correct competitiveness of the assay and to achieve the best LOD and working range of the immunosensor. To determine the optimal immunoreagents concentration, some BSA-CNH concentrations of 1, 10, and 100 μg/mL were immobilized on the sensor surface. MAb solutions of different concentrations in PBS were assayed with a flow rate of 20 μL/min. Under these conditions, maximum phase voltages were produced after 15 min of sample injection.

**Figure 9.**
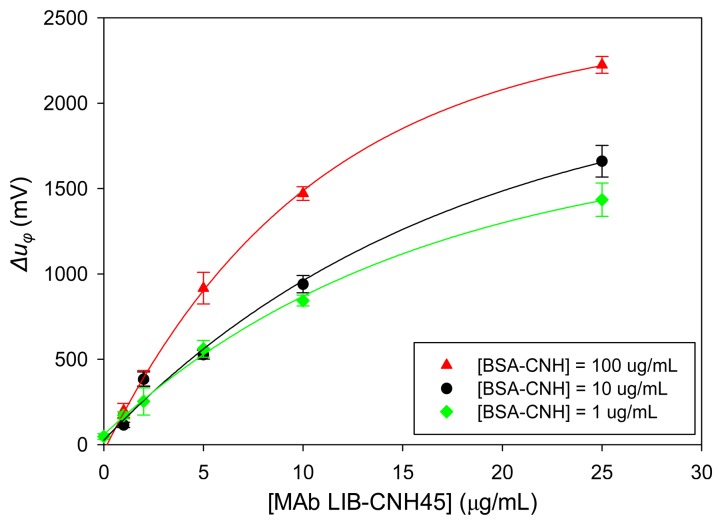
Optimization of the carbaryl LW immunosensor assay. Signal variation (Δ*u_φ_* after 15 min of sample injection at 20 μL/min) as a function of LIB-CNH45 MAb concentration. Triangles for the hapten-conjugate of 100 μg/mL; circles for the hapten-conjugate of 10 μg/mL; and diamonds for the hapten-conjugate of 1 μg/mL.

Figure 9 displays the phase signal changes (Δ*u_φ_*) obtained for LIB-CNH45 MAb assayed in the 1–25 μg/mL concentration range for the three different hapten-conjugate concentrations previously mentioned. As can be observed in the figure, the phase voltage did not reach an asymptotic maximum (plateau value), as usually occurs in these dose-responses curves. For the 10 μg/mL BSA-CNH concentration, higher concentrations of MAb were assayed and it was found that the plateau value was around a 50 μg/mL concentration of LIB-CNH45 that produced a Δ*u_φ_* of around 1971 mV (data not shown in Figure 9). For the rest of BSA-CNH concentrations, this plateau value was not sought to avoid excessive MAb waste. Moreover, for MAb concentrations of 2 μg/mL and above, the obtained signals were sufficient to distinguish them from noise and to work comfortably. In this way, the assayed values were sufficient to determine the optimal immunoreagents concentrations for a commercially attractive and sensitive immunosensor, where low immunoreagents concentrations are desired. It is well known that the lower concentration of employed immunoreagents, the better immunosensor sensitivity and LOD will be achieved [1].

In order to select the optimal hapten-conjugate concentration, the following criterion was considered: the lowest hapten-conjugate concentration, which leads to a high enough signal to distinguish it from noise. Following this criterion, the 100 μg/mL hapten-conjugate concentration was dismissed.

In relation to the optimal MAb concentration to guarantee a successful competitive immunoassay, a MAb concentration that provides less of the 60% of the obtained plateau value should be used. In our case, since the plateau value was at 50 μg/mL MAb concentration, this value was used to calculate the competitive conditions. Thus, MAb concentrations <10 μg/mL were determined as suitable for both selected BSA-CNH concentrations (1 and 10 μg/mL). To provide reasonable signals with a minimum MAb wasting, two preliminary MAb concentrations of 5 μg/mL and 2 μg/mL were chosen for the immunoassays.

### 3.3. Standard Calibration Curves and Assay Sensitivity

Several standard curves employing the selected immunoreagent concentrations (1 and 10 μg/mL for BSA-CNH conjugate and 2 and 5 μg/mL for LIB-CNH45) were performed.

**Figure 10.**
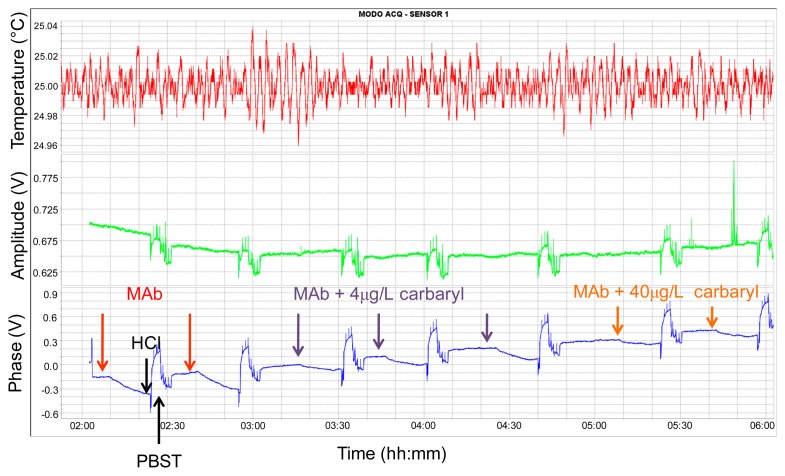
Real-time LW immunosensor response to analyte concentrations obtained with the AW-BIO v1.8 Software (AWSensors). Lower record: real-time phase voltage monitoring of consecutive carbaryl immunoassays. Increasing carbaryl concentrations were assayed with a constant LIB-CNH45 concentration of 2 μg/mL and a BSA-CNH concentration of 10 μg/mL. The regeneration (0.1 M HCl + PBST) is indicated whit a black arrow only for the first assay. Middle record: real-time monitoring of amplitude voltage in the same experiment. Upper record: real-time monitoring of the temperature during the experiment.

Figure 10 depicts a representative real-time record displayed by the software when some consecutive carbaryl concentrations were assayed during the development of a standard calibration curve experiment. In this figure, immunoassays were performed with a sensor immobilized with a BSA-CNH concentration of 10 μg/mL. Increasing carbaryl concentrations were assayed with a constant LIB-CNH45 concentration of 2 μg/mL and the sensor response to the different analyte concentrations was monitored. The phase voltage increments, Δ*u_φ_*, were used to generate the standard calibration curves. The regeneration event (0.1 M HCl + PBST) is indicated with a black arrow only for the first assay. At the same time, amplitude changes were recorded and even if these changes were much lower compared to phase changes, these data were useful to analyze the viscous losses and conformational properties of the interface layer, as will be seen later on.

The phase baseline signal presented a noise of around 3 mV. Such a noise value was estimated by calculating the standard deviation of the acquired data during 5 min previous to sample injection. In Figure 10, it can be appreciated that after regeneration of the sensing area, the baseline signal did not return to the desired initial phase voltage. Among others, two factors might have caused this unwanted effect: (1) One factor could be related to conformational changes in the immobilized layer during regeneration of each assay cycle. (2) Other possibility, also related to the regeneration of the sensing area, could be the fact that regeneration was not carried out effectively due to the shape of the employed rectangular seal. In addition, with the passing of time, it was observed that bubbles gathered in the corners of the PDMS rectangular seal. Therefore, we believe that the flow-through cell could be improved using other seal shapes or sealing approaches with no such abrupt corners, which, in our opinion, could significantly improve the immunosensor performance and could minimize the baseline drift. However, in spite of the baseline drift, the same phase increments were observed for the same carbaryl concentrations for every assay.

**Figure 11.**
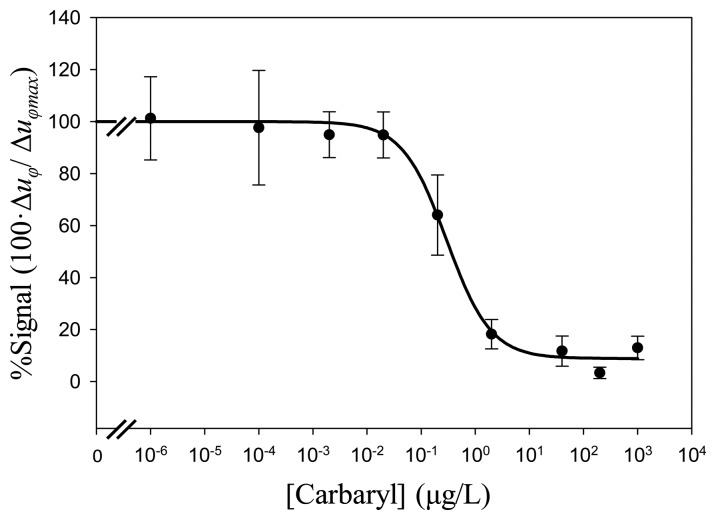
Standard calibration curve of carbaryl for a MAb concentration of 2 μg/mL and hapten-conjugate concentration of 10 μg/mL (Curve 2).

Figure 11 presents the standard calibration built up from the results produced in the experiment represented in Figure 10. Such a curve is the mean of several assays obtained with four different LW sensors. The analytical parameters of all the standard calibration curves performed in this work are summarized in Table 1 and the rest of the standard curves can be found in reference [11].

The comparison of Curves 1 and 2, both with a BSA-CNH concentration of 10 μg/mL, indicates that higher MAb concentrations lead, in principle, to worst immunosensor performances [1]. This confirms what it is stated in literature regarding the effects of the antibody concentration on the immunoassay sensitivity: for the same hapten-conjugate concentration, higher concentrations of antibody, despite rendering higher responses, usually tend to diminish the overall sensitivity of the assay [1]. Therefore, 5 μg/mL MAb concentration was not further assayed. Thus, the best combination, turned to be the one of Curve 2, presented in Figure 11. As expected for binding inhibition immunoassays, the calibration curve of Figure 11 showed the typical decreasing sigmoidal shape, *i.e.*, the signals provided by the piezoelectric immunosensor decreased as the analyte concentrations increased. The analytical parameters were: *I*_50_ value, which is generally accepted as the estimation of the immunosensor sensitivity, was 0.31 μg/L; the immunosensor limit of detection (LOD), calculated as the pesticide concentration producing 10% inhibition of the maximum signal (*I*_90_ value), was 0.09 μg/L, and the quantification range (linear working range, in which analyte concentrations produced signals between 80% and 20% inhibition of the maximum signal) was between 0.14 and 1.63 μg/L.

The standard calibration curves performed were not completely satisfactory for us, since the highest carbaryl concentrations did not produce the expected signal inhibition (at this concentrations a sensor signal of 0% was expected). To give an explanation to this phenomenon two hypotheses were established: (1) That after the immobilization of the sensors some spaces are left in the device surface where no BSA-CNH is immobilized. Thus, in these spaces antibodies can be trapped in a non-specific way producing a change in the phase-shift voltage signal. This phenomenon, might occur for all carbaryl concentrations. When the maximum signal that is used to calculate the relative measurements is lower, this effect is seen as stronger while, actually, it is not. (2) Another possible explanation of this phenomenon is that a high viscosity of the sample medium is produced when mixing the sample with high carbaryl concentrations. In the same way than hypothesis (1), this becomes an issue when the maximum signal is lower.

In order to check the veracity of hypothesis (1) a change in the previous established protocol was tested passing a 0.1% BSA solution with PBST through the sensor surface before injecting the sample with a high carbaryl concentration (200 μg/L). This was expected to block the empty spaces in the absence of hapten-conjugate at the interface layer avoiding non-specific interactions. The result was that BSA-PBST diminished this effect. For that reason, for future assays it is suggested to change the immunoassays protocol using BSA-PBST as the working buffer solution instead of only PBST. However, this change in the protocol did not completely solve the problem; thus, hypothesis (2) was also considered.

**Figure 12.**
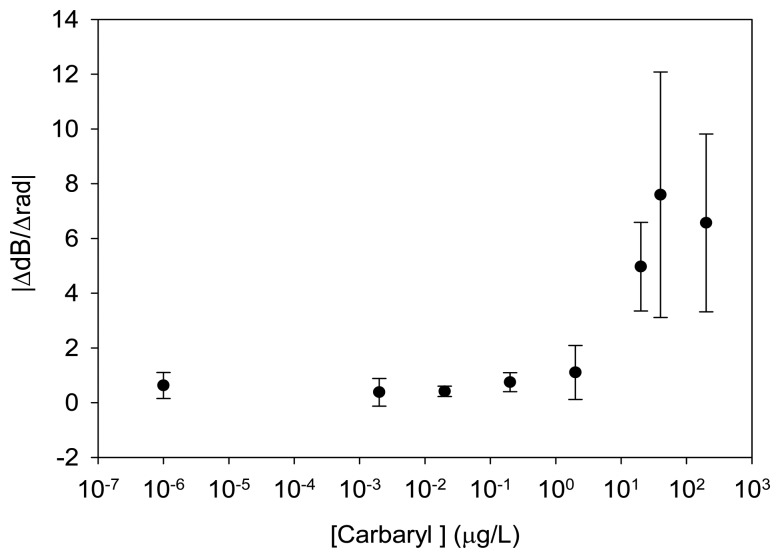
Acoustic ratios for the different carbaryl standards for a MAb concentration of 2 μg/mL and hapten-conjugate concentration of 10 μg/mL.

With the purpose of analyzing the viscoelastic behavior of the layer in touch with the sensing area of the immunosensor and explore the veracity of hypothesis (2), an additional study was performed calculating the ratio of the measured attenuation change and phase change. The amplitude to phase ratio (*acoustic ratio*, ΔdB/Δrad) of LW devices is a significant indicator of rigid or viscous interactions of the layer in contact with the sensor's vibrating surface [15,16]. A small ratio (<0.15 dB/rad in absolute value) occurs for a rigid loading, indicating that the attenuation-shift is small compared to the phase-shifts of the signal for such loading, while a high ratio (>8.8 dB/rad in absolute value) occurs for a viscous loading [12]. The acoustic ratios for the different carbaryl standard assays of Curve 2 were calculated and are presented in Figure 12. A higher acoustic ratio is observed for higher carbaryl concentrations, finding a maximum absolute value of 8 dB/rad. Therefore, a predominant viscous behavior of the layers in contact with the sensor can be considered for high carbaryl concentrations. Two different causes might be producing this effect: (1) the fluid medium on top of the sensor is changing its viscosity due to the high analyte concentration; or (2) the viscosity of the coating layer (hapten-conjugate layer) is changing because some spaces in the layer exist and are being filled with the high viscous medium, producing a mixed layer.

### 3.4. Analytical Performance Comparison between Different Carbaryl Detection Technologies

The analytical parameters for the detection of carbaryl obtained with different techniques, acoustic and non-acoustic, are summarized in Table 2. On the basis of these analytical parameters, the LW immunosensor is more sensitive (around ten-fold) than the one reported for the same compound using the surface plasmon resonance (SPR) transduction principle [17], where nearly the same immunoreagents were employed. However, it is still one order of magnitude less sensitive than the ELISA's one [18]. It is also important to mention that this ELISA employed a different conjugate-MAb pair, which provided higher signal responses than BSA-CNH-LIB-CNH45 pair, but does not allow regeneration [1]. Regeneration is an important feature of the implemented immunosensor that we were seeking, given that this characteristic makes an immunosensor more economical. Another important fact to mention is that this reported ELISA was optimized by changing some physicochemical factors on the analytical conditions of the immunoassay, such as pH, ionic strength, reagent concentrations, incubation times, and presence of Tween 20, which enhanced the sensitivity with respect to the non-optimized immunoassay [1,10]. Thus, the performance of the presented LW immunosensor could be further optimized in the same way.

In relation to the acoustic techniques, included in Table 2, the reported values correspond to experiments in which exactly the same immunoreagents were employed than the ones utilized for the developed LW immunosensor. Nevertheless, the characterization system and the measurement conditions were different for some of the technologies. In the case of the 9 MHz QCM immunosensor, an oscillator approach was used as the characterization technique and thus, frequency shifts (Δ*f* in Table 2) were measured [2]. In addition, they used a different flow-through system with a different flow-through cell, no thermostatic control and a different control and communications system. Therefore, LW immunosensor's analytical parameters turned to be around two orders of magnitude lower than those of the conventional QCM immunosensor. Hence, in our opinion, a considerable improvement has been achieved in this work.

In the case of 10 MHz QCM [19], a characterization technique based on the same approach than the one used in this work (phase measurements, Δ*u_φ_*) was employed, as well as the same immunoreagents. Nevertheless, the thermostatic and flow-through systems were different. As it can be inferred from Table 2, LW immunosensor's sensitivity and LOD also turned to be better than those of the 10 MHz QCM, around 50 times lower values than the conventional 10 MHz QCM.

The European Union has defined the limit concentration allowable in drinking water for each individual pesticide to 0.10 μg/L in order to protect human health (Drinking Water Directive 98/83/EC, 1998). Therefore, the analytical performance achieved by the reported LW immunosensor approaches this level and let us think that this LW device would be able to detect the carbaryl compound in water intended for human consumption at European regulatory levels in the near future. Nevertheless, some optimizations of the microsystem can be performed to further improve the performance of this immunosensor for the previously mentioned and other biosensing applications.

## 4. Conclusions

A custom-made microsystem for LW biosensing was developed, which included custom-made LW sensors and a flow-through cell for the sensors. An electronic characterization technique, recently proposed and validated for QCM devices, which is based on phase and amplitude detection at fixed frequency, was adapted for LW sensors. Immunosensors experiments were performed using the A10 Research Platform (AWSensors Corp., Spain), which integrated the characterization technique and the flow-through system. A LW-based immunosensor for the determination of carbaryl pesticide was developed with the platform. The immunosensor was based on a covalent hapten-conjugate immobilization and the use of monoclonal antibodies. The immunosensor achieved values of sensitivity (*I*_50_ value) and LOD (*I*_90_ value) of 0.31 μg/L and 0.09 μg/L, respectively, and its linear working range (*I*_80_–*I*_20_) was of 0.14–1.63 μg/L. The reported LW immunosensor was around one order of magnitude more sensitive than a SPR immunosensor developed for the same analyte under similar conditions. With some optimizations, the LW immunosensor would allow the determination of carbaryl in drinking water at European regulatory levels in the near future. These optimizations could include efforts in improving the sensor cell, changing some physicochemical factors of the immunoassays and/or testing lower immunoreagent concentrations. Nevertheless, with the results obtained so far, we think that LW immunosensors represent an economical alternative to other detection technologies and a significant promise in terms of simplicity of use and portability for on-line analysis.

## Figures and Tables

**Table 1.** Analytical parameters obtained from the curves of the different hapten-conjugate and monoclonal antibody (MAb) concentration combinations.

**Standard Curve**	**[BSA-CNH] (μg/mL)**	**[MAb] (μg/mL)**	**LOD, *I*_90_ (μg/L)**	**Sensitivity, *I*_50_ (μg/L)**	**Working Range (μg/L)**
Curve 1	10	5	0.44	2.78	0.86–8.69
Curve 2	10	2	0.09	0.31	0.14–1.63
Curve 3	1	2	0.14	1.40	0.33–5.98

**Table 2.** Analytical parameters of carbaryl determination based on ELISA, SPR, Quartz Crystal Microbalance (QCM) and the developed Love Wave (LW) immunosensor. Δ*f* means that the measurement were carried out using an oscillator electronic configuration, and Δ*u_φ_* means that the measurement were carried out using the described phase and amplitude detection at fixed frequency.

**Technique**	**Sensitivity, *I*_50_ (μg/L)**	**LOD, *I*_90_ (μg/L)**	**Working Range (μg/L)**
ELISA [18]	0.06	0.01	0.02–0.18
SPR [17]	3.11	1.38	1.90–6.34
QCM 9 MHz (Δ*f*) [2]	30.00	11.00	15.00–53.00
QCM 10 MHz (Δ*u_φ_*) [19]	16.70	4.00	7.00–35.00
LW 120 MHz (Δ*u_φ_*)	0.31	0.09	0.14–1.63
